# iTRAQ-based proteomic profiling of salt-tolerant and salt-sensitive potato (*Solanum tuberosum*) cultivars under salinity stress

**DOI:** 10.3389/fpls.2025.1633048

**Published:** 2025-12-01

**Authors:** Zhaojuan Zhang, Yongsheng Wang, Shuai Zhao, Fangyu Chen, Rongyu Huang, Tiancang Na, Yuchun Guo

**Affiliations:** 1 Key Laboratory of Ministry of Education for Genetics, Breeding and Multiple Utilization of Crops, College of Agriculture, Fujian Agriculture and Forestry University, Fuzhou, China; 2 Key Laboratory of Ministry of Agriculture and Rural Affair for Biological Breeding for Fujian and Taiwan Crops, College of Agriculture, Fujian Agriculture and Forestry University, Fuzhou, China; 3 Fujian Provincial Key Laboratory of Crop Breeding by Design, College of Agriculture, Fujian Agriculture and Forestry University, Fuzhou, China; 4 School of Computer Science and Information Engineering, Fuyang Normal University, Fuyang, Anhui, China; 5 College of Life Science, Fujian Agriculture and Forestry University, Fuzhou, Fujian, China; 6 School of Life Sciences, Xiamen University, Xiamen, Fujian, China; 7 Academy of Agriculture and Forestry Sciences, Qinghai University, Xining, Qinghai, China

**Keywords:** *Solanum tuberosum*, soil salinity, iTRAQ-based quantitative proteomics, differentially expressed proteins, protein-protein interaction

## Abstract

**Introduction:**

Soil salinity represents a significant abiotic stress factor that adversely affects potato yield and quality. Elucidating the molecular mechanisms underlying salt tolerance is crucial for the development of resilient cultivars. This study examines the proteomic responses of salt-tolerant (M5008) and salt-sensitive (D516) potato cultivars under saline conditions.

**Methods:**

A quantitative iTRAQ-based proteomic approach was utilized to analyze protein expression profiles in the roots of both cultivars exposed to 150 mM NaCl stress. Bioinformatics analyses—including Gene Ontology (GO) annotation, Kyoto Encyclopedia of Genes and Genomes (KEGG) pathway enrichment, and protein–protein interaction (PPI) network construction—were performed. Key results were further validated by quantitative real-time PCR (qRT-PCR).

**Results:**

A total of 511 and 456 differentially accumulated proteins (DAPs) were identified in D516 and M5008, respectively. These DAPs were predominantly involved in redox homeostasis, sugar and osmotic metabolism, and phytohormone signaling pathways. PPI network analysis revealed six major functional modules, including glucose metabolism, translational initiation, and ubiquitin-mediated protein catabolism. The expression patterns of key proteins (G6PD1, P5CSA, PP2A2, TPS1, GAPCP1, HEXO1) were consistent with their corresponding mRNA levels, supporting their functional roles in the salt stress response.

**Discussion:**

The salt-tolerant cultivar M5008 demonstrates a coordinated and multifaceted response to salinity stress, characterized by enhanced antioxidant defense, efficient energy utilization, and precise regulation of protein synthesis and degradation. In contrast, the salt-sensitive cultivar D516 exhibits a disorganized and less effective response. These findings offer new insights into the proteomic mechanisms governing salt tolerance in potato and identify potential candidate genes for use in future breeding and genetic engineering efforts.

## 1 Introduction

Soil salinization is one of the most widespread abiotic stresses affecting the growth, distribution, and productivity of many crop species. It is estimated that approximately 20% of irrigated land is threatened by salinity (Dos Santos et al., 2021; Balasubramaniam et al., 2023). Potato (*Solanum tuberosum*) plays a crucial role in securing the global food supply (Devaux et al., 2021). Although many potato cultivars were proven to be moderately sensitive to salinity, the salt tolerance was different among the potato cultivars (Sobhanian et al., 2011). Therefore, understanding the salt tolerance-associated mechanisms and genetic improvement of salinity stress tolerance would help address concerns in land utilization and crop production.

Salt stress retards plant growth and development, including osmotic stress, ionic imbalance, and oxidative stress (Aslam et al., 2019; Hasanuzzaman et al., 2021; Fu and Yang, 2023). A high level of NaCl in plant cells perturbs the ion equilibrium, leading to K^+^ deficiency and excessive Na^+^ accumulation (Han et al., 2015). To survive, plants have evolved many strategies, including ion-selective absorption or excretion and ion compartmentalization (Han et al., 2015; Peng et al., 2016). The identified genes associated with Na^+^ excretion and intracellular compartmentalization, such as *AtNHX1*, *AtSOS1*, and *AtSOS2*, shed light on the mechanisms by which plants maintain ion balance under salt stress (Abbasi-Vineh et al., 2021; Dzinyela et al., 2023). During the salt stress response, reactive oxygen species (ROS) function as crucial secondary messengers to activate downstream pathways (Tomar et al., 2021; Kesawat et al., 2023). Furthermore, salt stress causes overproduction of ROS, leading to severe detrimental effects on DNA, lipids, and fundamental cellular structures (Ahmad et al., 2019; Aslam et al., 2019; Hasanuzzaman et al., 2021; Tomar et al., 2021; Kesawat et al., 2023). To deal with oxidative stress, redox homeostasis in salt-tolerance species is maintained by two strategies, including non-enzymatic antioxidant compounds (such as glutathione, ascorbate, and tocopherols) and antioxidant enzymes (such as superoxide dismutase (SOD), glutathione reductase (GR), and ascorbate peroxidase (APX)) (Hasanuzzaman et al., 2021; Massange-Sánchez et al., 2021). Therefore, an effective strategy for minizine salt stress should be taken into consideration in developing a successful tool for salt tolerance mechanisms.

The iTRAQ (isobaric tags for relative and absolute quantification) technique combined with LC-MS (liquid chromatography-tandem mass spectrometry)/MS has been implemented for the identification of key mechanisms responsible for salt tolerance due to the advantages of high resolution and precise quantification (Ross et al., 2004). In addition, this technology has been used to analyze rice (Lakra et al., 2019), cotton (Yuan et al., 2023), maize (Weng et al., 2021), and cucumber (Fan et al., 2015). Combined with bioinformatics technology, the complex mechanisms of response to salt stress in the above species have been revealed by the expanded proteomics strategies. However, comprehensive proteomic analyses of diverse potato cultivars under salt stress conditions remain limited. Systematic investigation of the proteomic dynamics between salt-tolerant and salt-sensitive potato cultivars can facilitate the identification of key protein networks and molecular mechanisms underlying salt tolerance. Therefore, mining genes associated with salt tolerance and elucidating the corresponding molecular mechanisms are essential for the development of salt-tolerant potato cultivars.

In the initial germplasm screening analysis, ‘M5008’ potatoes were regarded as salt-tolerant, while ‘D156’ potatoes were identified as salt-sensitive. In this study, an iTRAQ-based quantitative proteomic approach was employed to analyze protein expression profiles in the roots of the salt-tolerant cultivar ‘M5008’ and the salt-sensitive cultivar ‘D516’ under salinity stress. The objectives of this research were: (1) to identify differentially accumulated proteins (DAPs) between these two contrasting cultivars in response to salt stress; (2) to perform Gene Ontology (GO) and Kyoto Encyclopedia of Genes and Genomes (KEGG) enrichment analyses to characterize the biological processes and metabolic pathways associated with the identified DAPs; (3) to construct protein–protein interaction networks for the identification of functionally significant modules; and (4) to validate the expression levels of key proteins using qRT-PCR. The results provide valuable insights into the molecular mechanisms underlying salt tolerance in potato and highlight potential candidate genes for the genetic improvement of salt-tolerant cultivars.

## 2 Materials and methods

### 2.1 Plant materials treatment and sample collection

Two potato cultivars, salt-tolerant (Min 08085008, M5008) and salt-sensitive (Zhongshu D516, D516), were used in this study. Prior to this study, these two culti-vars were screening from 18 candidate cultivars (including Long 201205-6, Zhongshu D516, Favorita, Guinongshu No.1, Zhongshu N88, A7, Yunshu 506, Zihua 851, Xingjia No.2, Zhongshu No.3, Huasong 66, Minshu No.1, Heijingang, 08CA0687, Min 08085008, Black Rose No.1, and Zhongshu 157) based on our results of saline test in Fuqing, Fuzhou, Fujian, China (25.55° N, 119.50° E, elevation 24 m) and 16 different saline regions test (4 locations in Fuqing, Fuzhou, Fujian, 3 locations in Changle dis-trict, Fuzhou, Fujian [25.88° N, 119.60° E], 5 locations in Puxia County, Ningde, Fujian [26.91° N, 120.22° E], 3 locations in Xiuyu district, Putian, Fujian [25.31° N, 119.22° E], and 1 location in Research Center, Fujian Agriculture and Forestry University [26.05° N, 119.13° E]) in 2015. Two potato cultivars were selected: a salt-tolerant variety (M5008) and a salt-sensitive variety (D516). Tubers of both cultivars were planted in a substrate mixture (vermiculite:perlite:nutrient soil = 1:1:1) and grown for one month in a greenhouse under a 16/8-hour light/dark cycle at 25°C. Seedlings of *uniform* growth (15–20 cm in height) were transplanted into 6-liter black containers filled with 5 liters of 1/20× Murashige & Skoog (MS) nutrient solution. The nutrient solution was renewed every two days. After seven days, morphologically similar seedlings were selected and transferred to a hydroponic system using perforated foam boards in containers, with water replenished every 24 hours. For each cultivar, uniformly grown seedlings were divided into two groups: the salt-treated group received a nutrient solution supplemented with 150 mmol/L NaCl, while the control group was maintained in 1/20× MS nutrient solution. Each treatment included three independent biological replicates, with each replicate consisting of a pooled sample of roots from six individual seedlings.Root samples were collected at 0, 12 hours post-treatment, immediately frozen in liquid nitrogen, and stored at -80°C for subsequent proteomic and transcriptomic analyses. In addition, fresh potato roots (0.2 g each) were chopped and placed into pre-cooled mortars. Then, 2 ml of 0.05 M PBS (pH 7.0, containing 1% PVP) buffer was added, and the tissues were ground under icebath conditions. The homogenate was transferred into a 10 ml centrifuge tube, and the mortar was rinsed twice with 3 ml of PBS buffer. The combined solution was centrifuged at 4°C and 4000 r·min^−1^ for 15 min. The resulting supernatant was stored at 4°C for subsequent assays of superoxide dismutase (SOD), peroxidase (POD), and catalase (CAT) activities. SOD activity was determined by colorimetric method, POD activity by the guaiacol method, CAT activity by ultraviolet absorption method, and malondialdehyde (MDA) content by thiobarbituric acid (TBA) colorimetric assay.

### 2.2 Protein preparation and iTRAQ labeling

Protein extraction was conducted using trichloroacetic acid/acetone precipitation with phenol extraction methods as described in a previous study (Wu et al., 2014). The roots (3.0 g) of potato seedlings treated with 0.15 M NaCl or without treatment for 12 h were collected and pulverized in mortar with liquid nitrogen and appropriate amount poly(vinylpolypyrrolidone) (PVPP). After adding 6 mL of protein extraction buffer (pH 8.0, containing 50 mM Tris-HCl, 25 mM EDTA, 0.5 M thiourea, and 0.5% (w/v) β-mercaptoethanol), the samples were transferred to a 50-mL tube, vortexed thoroughly, and let stand for 1 h at 4°C, followed by centrifugation at 15,000 *g* for 15 min at 4°C. The supernatant was removed to a new tube and precipitated by added 7–8 volumes of cooled acetone (pH 8.0, containing 0.07% (w/v) β-mercaptoethanol) and incubated at -20°C overnight. The precipitate was collected by centrifugation at 15,000 *g* for 8 min at 4°C, and washed twice with 10 mL of acetone, followed by dissolved in 5 mL of 0.1 M Tris-HCl buffer (pH 8.0, containing 100 mM Tris-HCl, 50 mM EDTA, and 2% (w/v) β-mercaptoethanol) for 10 min at 4°C, and extracted by adding 5 mL of Tris-phenol for 15 min under continuous shaking at room temperature. The phenol phase was separated by centrifugation at 15,000 *g* for 10 min at 4°C, and precipitated by 7–8 volumes of a 0.1 M ammonium acetate/methanol solution, incubated at -20°C overnight. The pellets were collected by centrifugation at 15,000 *g* for 10 min at 4°C, and then washed 2–3 times by using a 10 mL 0.1 M ammonium acetate/methanol solution, and again 2–3 times by adding 10 mL of acetone. The resulting pellets were dried under vacuum, dissolved in lysis buffer (containing 7 M urea, 2 M thiourea, 4% (w/v) CHAPS, and 40 mM DL-dithiothreitol (DTT)), Protein concentration was measured accurately using a 2D Quant Kit (GE, USA) and was adjusted to 1 μg·μL^-1^ with lysis buffer. Then, 100 μL of the protein sample was transferred to a new tube, 400 μL of methyl alcohol was added, and the mixture was carefully vortexed. Afterward, 100 μL chloroform and 300 μL Milli-Q water were added and mixed with a vortex. The sample was centrifuged for 10 min at 20,000 g and 4°C, and the top organic phase was removed carefully. Next, 300 μL methyl alcohol was added and mixed carefully by vortexing. Proteins were finally precipitated by centrifugation (10 min, 20,000 g at 4°C), and the precipitate was vacuum-dried. The protein pellet was dissolved in 20 μL of 50 mM NH_4_HCO_3_ at room temperature and subsequently reduced in 50 mM DTT for 30 min at 56°C. After cooling to room temperature, the sample was alkylated by incubating in 50 mM iodoacetamide for 30 min in the dark. After adding 50 μL 100 mM NH_4_HCO_3_, 10 μL 0.1μg·μL^-1^ trypsin (Promega, USA) was added to the protein sample. The protein sample was digested by incubation at 37°C on a rocking shaker for 16 h in the dark, and 10 μL 20% formic acid was added to acidize the sample. Sample desalination was performed by Sep-Pak C18 columns (Waters, USA), and the samples were washed with 300 μL Milli-Q water and eluted in 50 μL methyl alcohol. The elution was dried under vacuum and stored at -80°C for further use. iTRAQ labelling of peptides was performed using an iTRAQ 4-plex kit (Applied Biosystems, USA) according to the manufacturer’s instructions. After the peptides were labelled with the isobaric tags, they were incubated at room temperature for 1 h. The peptide mixtures of different samples were subsequently pooled, dried by vacuum centrifugation and stored at -80°C until LC-MS/MS analysis were performed, and then stored at -80°C. A total of 100 µg of proteins were reduced, alkylated, trypsin digested, and labeled with iTRAQ regents (Applied Biosystems, USA) according to the manufacturer’s instructions.

### 2.3 LC-MS/MS

The LC-MS/MS and data analysis of the peptides were performed on a TripleTOF 5600 MS (AB SCIEX, Concord, ON, CA, USA) according to the method as described by Zhong et al. (2014). Briefly, the dried peptides were dissolved in 2% ACN containing 0.1% FA and analyzed. Through a Nanospray III source (AB SCIEX), a splitless Ultra 2D Plus (Eksigent, Dublin, CA, USA) system was coupled to the TripleTOF 5600. HPLC gradients were delivered at 300 nL/min, and 60-min gradient was employed for each peptide fraction. Peptides were separated on a fused silica capillary emitter (New Objective, Woburn, MA, USA) packed in-house with 5 m C18 resin, and analyzed in the positive ion mode by ESI (spray voltage =2.4 kV). Survey scans were acquired in 250 ms and 20 product ion scans were collected in 50 ms per scan. For obtaining better iTRAQ reporter ion signal, adjust collision energy (adjust CE) was selected. The data have been submitted to the iProX platform and are available for sharing through this repository. The PXD accession number is PXD068921.

### 2.4 Database search, protein identification, and quantification

**Figure 1:**
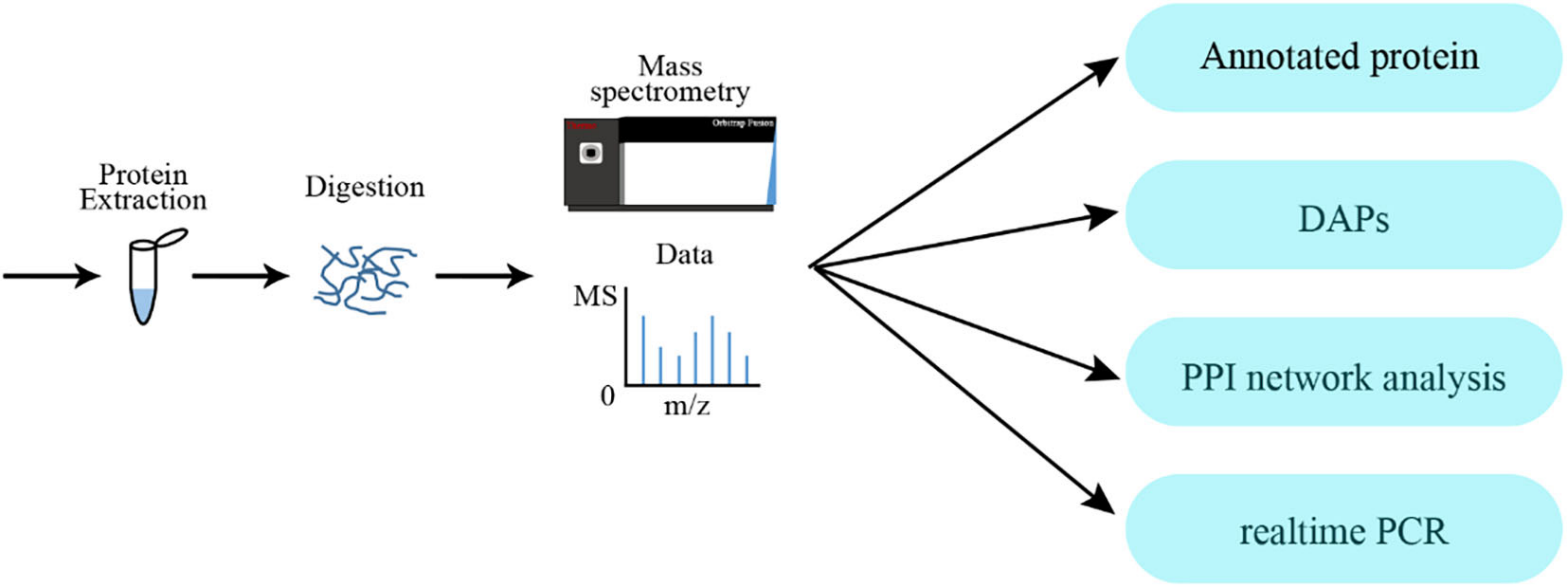
Experimental strategy and annotation of proteome analysis.

The overall experimental strategy for proteomic analysis is illustrated in **Figure 1**. To identify and quantify the protein in ‘D516’ and ‘M5008’ cultivars, the results of MS/MS data were searched against the *Solanum tuberosum* from the Uniport database (Bairoch and Apweiler, 2000) using MaxQuant (Tyanova et al., 2016). The specified cleavage enzyme and false discovery rate (FDR) were set as trypsin/P and 0.01, respectively. Other parameters, such as mass error for precursor ions and fragment ions, fixed modifications, variable modifications, and minimum peptide length, were set as default values. Proteins with at least one unique peptide were retained for further quantification and analysis. iTRAQ-plex was selected for the ‘D516’ and ‘M5008’ samples as the quantification method.

### 2.5 Bioinformatics analysis

In this study, all identified proteins were blasted into known public databases, such as COG, GO, KEGG, KOG, Pfam, Swissport, TrEMBL, and NR (Ashburner et al., 2000; Bairoch and Apweiler, 2000; Tatusov et al., 2000; Kanehisa et al., 2002; Pruitt et al., 2007; El-Gebali et al., 2019) to obtain the detail function and annotation for further analysis. Differentially expressed proteins were identified using a combination fold change (≥ 1.5 or ≤ 0.66) and P values (< 0.05) calculated using the DESeq R package (Anders and Huber, 2010). GO enrichment analysis and KEGG pathway enrichment analysis were performed using the bingo of Cytoscape (Maere et al., 2005) and cluster profiler (Yu et al., 2012). The interaction network of proteins was obtained by the STRING database (http://string-db.org/), and then the functional cluster of the network was identified by the MCODE (Bader and Hogue, 2003) of Cytoscape.

### 2.6 Quantitative real-time PCR validation of proteomic

Total RNA was extracted from potato roots treated with either 150 mM NaCl or water (control) for 12 hours using the Tiangen RNA extraction regent kit according to the manufacturer’s instructions. cDNA synthesis was performed using the PrimerScript™ RT Reagent Kit with gDNA Eraser (Takara, Shiga, Japan). qRT-PCR was performed using the SYBER premix ExTaq™ Kit (Takara, Shiga, Japan) and the 7500 Real-Time PCR system (Applied Biosystems). Gene-specific primers (listed in **Supplementary Table S5**) and SYBR Premix EX Taq. Actin was applied as an internal reference gene. It was used as the reference gene. Three replicates per treatment were performed.

## 3 Result

### 3.1 ‘D516’ and ‘M5008’ cultivars showed distinct phenotypes with respect to salt tolerance

**Figure 2:**
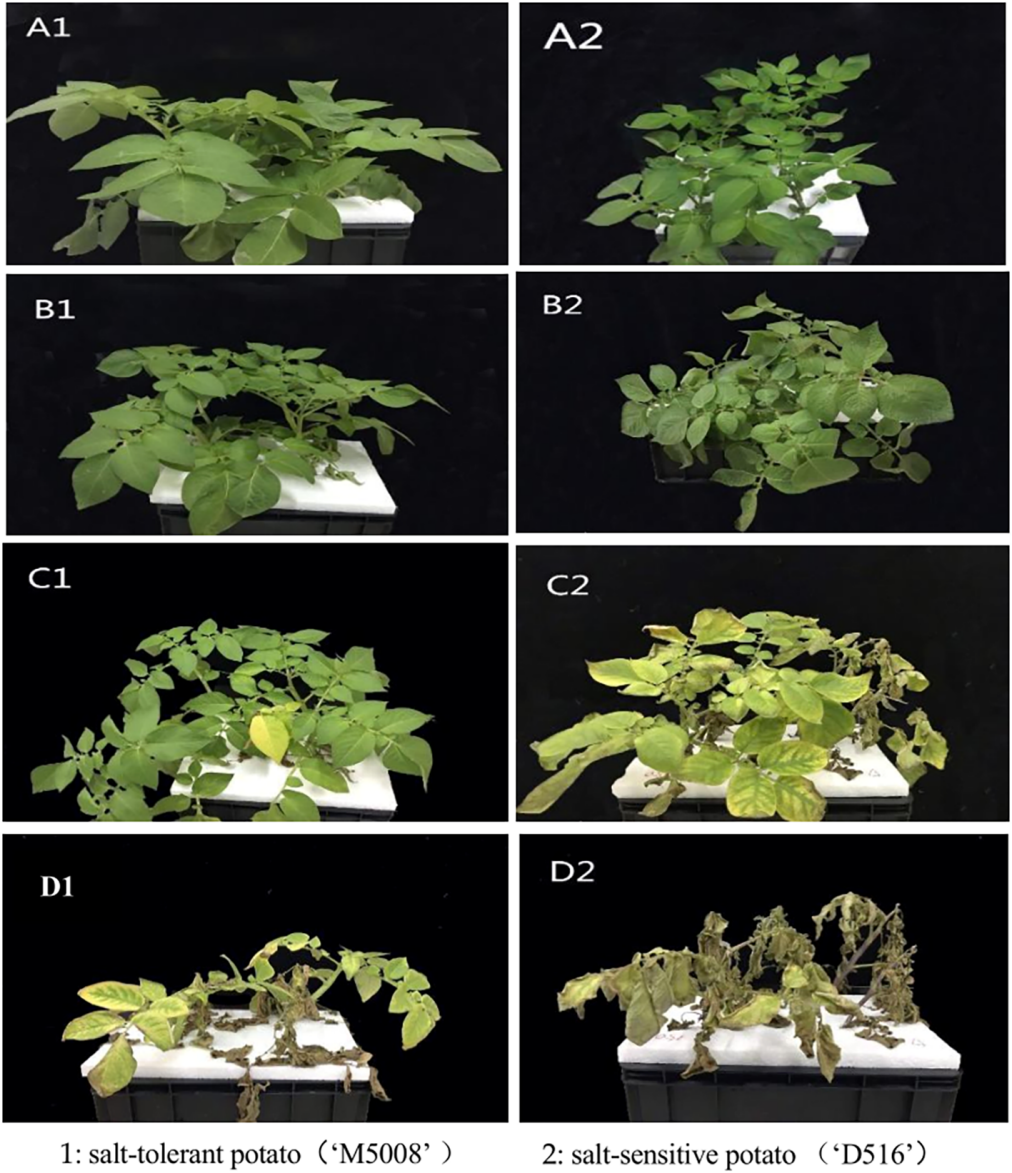
Morphological manifestations of various salt-tolerant potato varieties under different stress time at salt concentrations of 150mM. Different letters represent different stress times: **(A)** 0h; **(B)** 12h; **(C)** 96h; **(D)** 168h.

As expected, the ‘M5008’ cultivar exhibited a higher salt resistance trait (**Figure 2**). Compared to the control, the leaves of ‘D516’ cultivar turned yellow while the leaves of ‘M5008’ cultivar had a stay-green phenotype at 12 h post salinity treatment.

**Figure 3:**
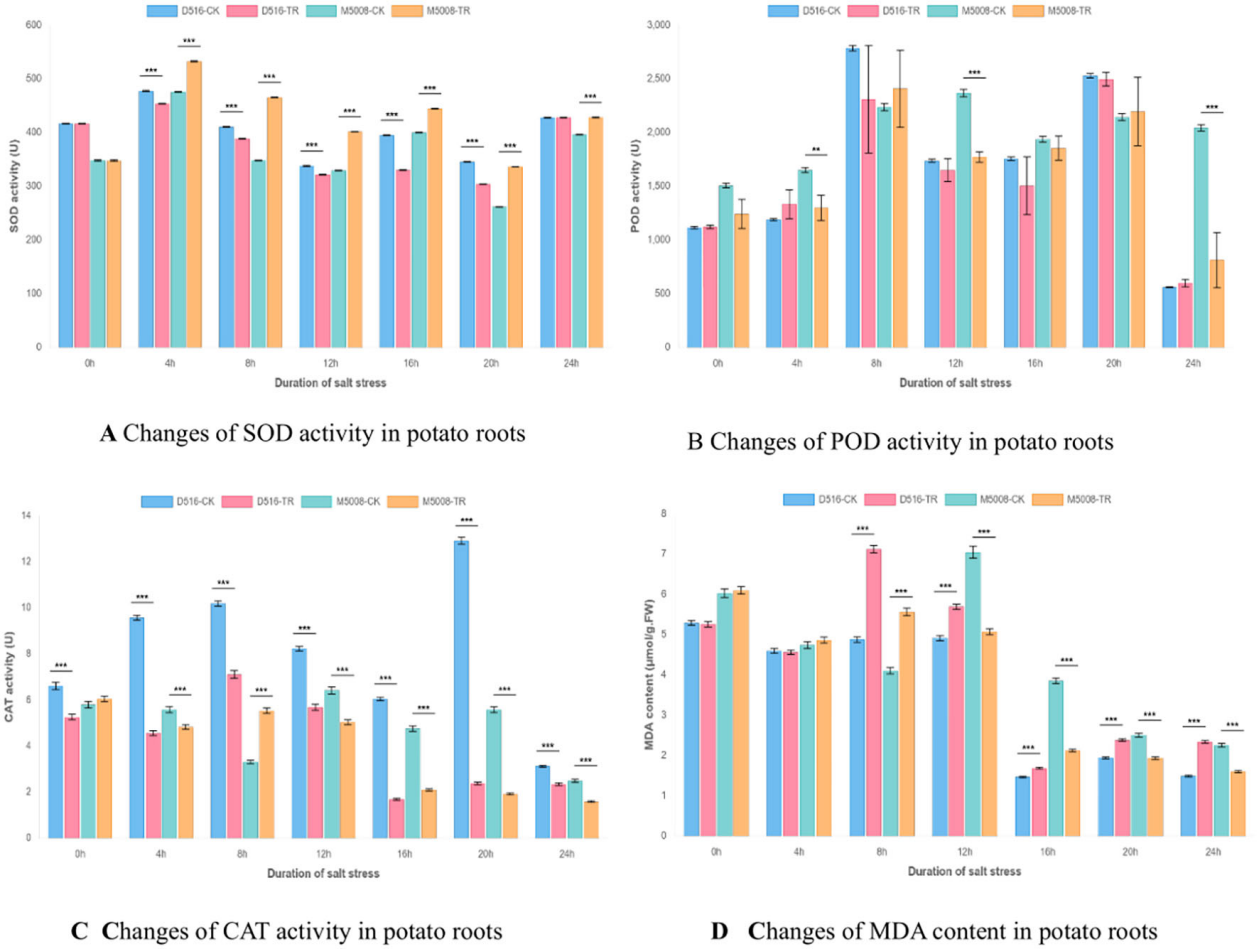
Dynamic changes in antioxidant enzyme activities and lipid peroxidation in potato cultivars under salt stress. **(A)** Superoxide dismutase (SOD) activity. **(B)** Peroxidase (POD) activity. **(C)** Catalase (CAT) activity. **(D)** Malondialdehyde (MDA) content. Data are presented for four treatments: D516 control (D516-CK), D516 salt-treated (D516-TR), M500B control (M500B-CK), and M500B salt-treated (M500B-TR). Significant differences between control and treated groups at the same time point are indicated by asterisks (*p < 0.05, **p < 0.01, ***p < 0.001).

As illustrated in **Figure 3**, the two potato varieties exhibited fundamentally distinct physiological responses to salt stress across all measured parameters. The salt-tolerant variety M5008 demonstrated a coordinated and robust antioxidant defense system, whereas the salt-sensitive variety D516 displayed progressive dysfunction in its oxidative stress management mechanisms.

Superoxide dismutase (SOD) activity revealed marked varietal differences (**Figure 3A**). M5008 showed significantly enhanced SOD activity during the 4–20 h stress period (p < 0.001), with a peak increase of 33.7% at 8 h (465.4 U in TR vs. 348.1 U in CK), indicating efficient scavenging of superoxide radicals. In contrast, D516 exhibited significant suppression of SOD activity between 8 and 20 h (p < 0.05–0.01), most pronounced at 20 h, where activity decreased by 12.0% (304.0 U in TR vs. 345.6 U in CK).

Analysis of peroxidase (POD) activity (**Figure 3B**) further highlighted inter-varietal differences in hydrogen peroxide detoxification capacity. M5008 displayed significantly elevated POD activity at 8–12 h (p < 0.05), particularly at 12 h (2367.1 U in TR vs. 1771.7 U in CK), supporting effective H_2_O_2_ clearance during critical phases of stress. In contrast, D516 exhibited inconsistent POD responses accompanied by high intra-group variability, most evident at 8 h, where TR values ranged from 1312.5 to 2877.7 U compared to the stable D516-CK value of 2784.4 U, suggesting inadequate regulatory control over this enzymatic pathway.

Catalase (CAT) activity profiles (**Figure 3C**) revealed the most pronounced divergence between varieties. M5008 maintained relatively stable CAT activity throughout the stress period, with transient upregulation during early stress (4–8 h), reflecting functional resilience. Conversely, D516 exhibited severe and sustained suppression of CAT activity across all time points (p < 0.001), with enzyme levels declining to only 14–18% of CK values at 16–20 h—indicative of a near-complete breakdown of this key antioxidant mechanism.

Measurements of malondialdehyde (MDA) content (**Figure 3D**) provided direct evidence of differential membrane integrity under salt stress. M5008 experienced only transient lipid peroxidation, with rapid recovery following an initial spike at 8 h (5.56 μmol/g FW in TR vs. 4.10 μmol/g FW in CK; p < 0.001). In contrast, D516 suffered extensive and persistent membrane damage, as reflected by a 45.9% increase in MDA at 8 h (7.12 μmol/g FW in TR vs. 4.10 μmol/g FW in CK; p < 0.001) and significantly elevated levels from 8 to 24 h (p < 0.01–0.001).

A comprehensive evaluation of these physiological parameters indicates that salt tolerance is primarily determined by the synergistic functionality of the antioxidant network rather than the performance of individual enzymes. In M5008, balanced coordination among SOD, POD, and CAT enables effective mitigation of reactive oxygen species (ROS) accumulation and preservation of cellular membrane integrity. In contrast, D516 exhibits a progressive failure of antioxidant defenses—initiated by impaired SOD activity, exacerbated by erratic POD regulation, and culminating in the collapse of CAT function—resulting in uncontrolled oxidative stress and irreversible membrane deterioration.

These physiological findings are consistent with and functionally validate the proteomic data presented in the main manuscript. The constitutively prepared cellular state and stable expression of ROS-scavenging enzymes in M5008 align with its robust and well-coordinated antioxidant response, enabling effective containment of oxidative damage. Conversely, D516’s high-amplitude but dysregulated induction of antioxidant enzymes proves insufficient to counteract progressive oxidative injury, underscoring fundamental differences in antioxidant strategy between salt-sensitive and salt-tolerant potato cultivars.

Therefore, the distinct salt tolerance between the two cultivars renders them ideal models for exploring the underlying mechanisms of potato salt tolerance. Then, three independent samples of D516 and M5008 were collected for protein sequencing using comparative iTRAQ analysis (**Table 1**), and a total of 4505 proteins were identified (**Supplementary Table S1**). These identified proteins were blasted into different databases to obtain the annotation. As indicated, most proteins (4498) had homologous proteins in the NR proteins database. Furthermore, 4125 (91%) proteins had significant matches in the GO database, and 2015 (45%) proteins had matches in the KEGG database.

**Table 1:** The annotation of all identified proteins in the two potato cultivars.

Annotated database	Annotated number	300 <=length<1000	Length>=1000
COG	2664	1652	82
GO	4125	2486	112
KEGG	2015	1222	70
KOG	3278	2020	89
Pfam	9075	6069	452
Swissprot	4249	2573	118
TrEMBL	4100	2449	112
Nr	4498	2693	124
All	4505	2695	124

**Figure 4:**
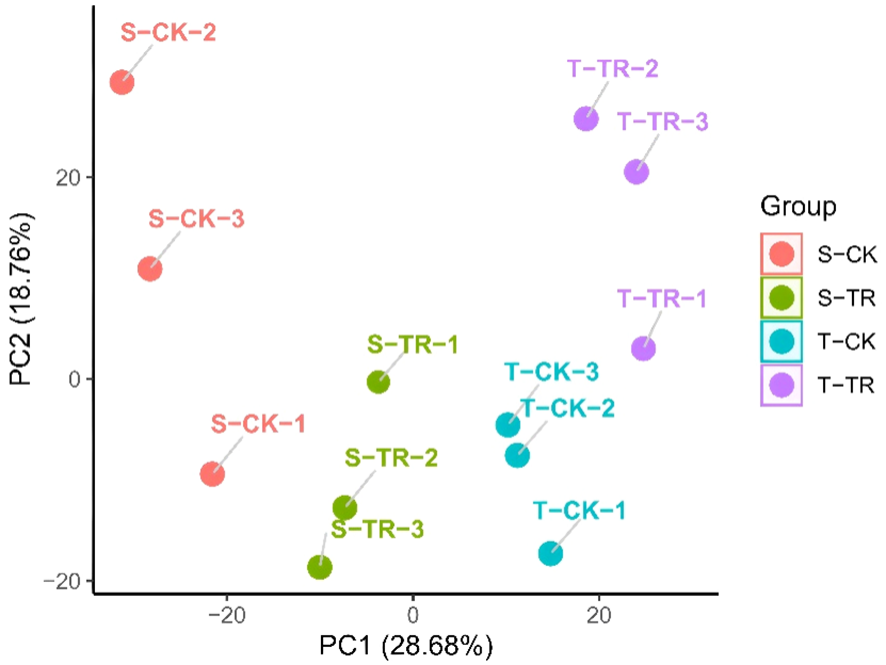
PCA of protein express data.

The results of the PCA (**Figure 4**) analysis indicate that the transcriptomic profiles of potato root samples are strongly influenced by both experimental treatment and cultivar-specific responses.

The tight clustering of the three biological replicates within each group (e.g., T-TR-1, 2, 3; S-TR-1, 2, 3) reflects high experimental reproducibility and data reliability. This consistent grouping validates the quality of the RNA-seq data and supports the robustness of the observed gene expression patterns.

The most prominent pattern is the clear separation of samples along the PC1 axis, which accounts for 28.68% of the total variance and primarily corresponds to the treatment effect (salt stress vs. control).

Samples on the right side of PC1 (positive values) include all control groups (CK), regardless of cultivar (S-CK and T-CK), which cluster closely together. This indicates that under non-stress conditions, the global gene expression profiles of the two cultivars are highly similar, with minimal baseline transcriptional divergence.

Conversely, all salt-stressed samples (TR), from both cultivars, are positioned on the left side of PC1 (negative values). This pronounced shift demonstrates that salt stress induces a substantial and genome-wide reprogramming of the transcriptome in potato roots. The magnitude of this change underscores that the imposition of salt stress is the primary source of variation in the dataset, overshadowing inherent genetic differences between the cultivars.

While treatment is the dominant factor, the second principal component (PC2), explaining 18.76% of the variance, captures key genotypic differences in the transcriptional response to stress.

The salt-sensitive ‘D516’ under stress (S-TR) is located in the bottom-left quadrant, with its three replicates forming a distinct cluster. In contrast, the salt-tolerant ‘M5008’ under stress (T-TR) occupies the top-left quadrant. The separation between the S-TR and T-TR groups along PC2 provides clear evidence that the two cultivars employ fundamentally different molecular strategies in response to the same stress stimulus. This differential regulation likely contributes to the superior physiological performance of ‘M5008’.

Notably, the T-TR group exhibits a more compact cluster compared to S-TR along both PC1 and PC2 axes. This tighter aggregation suggests that the transcriptional response in the tolerant cultivar is more consistent, coordinated, and tightly regulated. In contrast, the relatively greater dispersion of the S-TR replicates may reflect a more variable, less synchronized, or potentially dysregulated response in the sensitive cultivar, which could underlie its reduced capacity to cope with salt stress.

Overall, the PCA plot reveals a two-tiered transcriptional response to salt stress in potato:Tier 1 – A Shared Stress Response (PC1): Both cultivars exhibit a strong, common transcriptional reaction to salt stress, clearly distinguishing stressed samples from controls. This represents a core stress-responsive program activated across genotypes.Tier 2 – Genotype-Specific Regulatory Divergence (PC2): Within this shared response framework, the two cultivars diverge significantly in their transcriptional reprogramming. The tolerant ‘M5008’ activates a distinct and well-coordinated set of genes—or modulates their expression to differing extents—constituting an effective adaptive mechanism that is either absent or inadequately executed in the sensitive ‘D516’.

### 3.2 Identification and functional annotation of DAPs between the two potato cultivars

**Figure 5:**
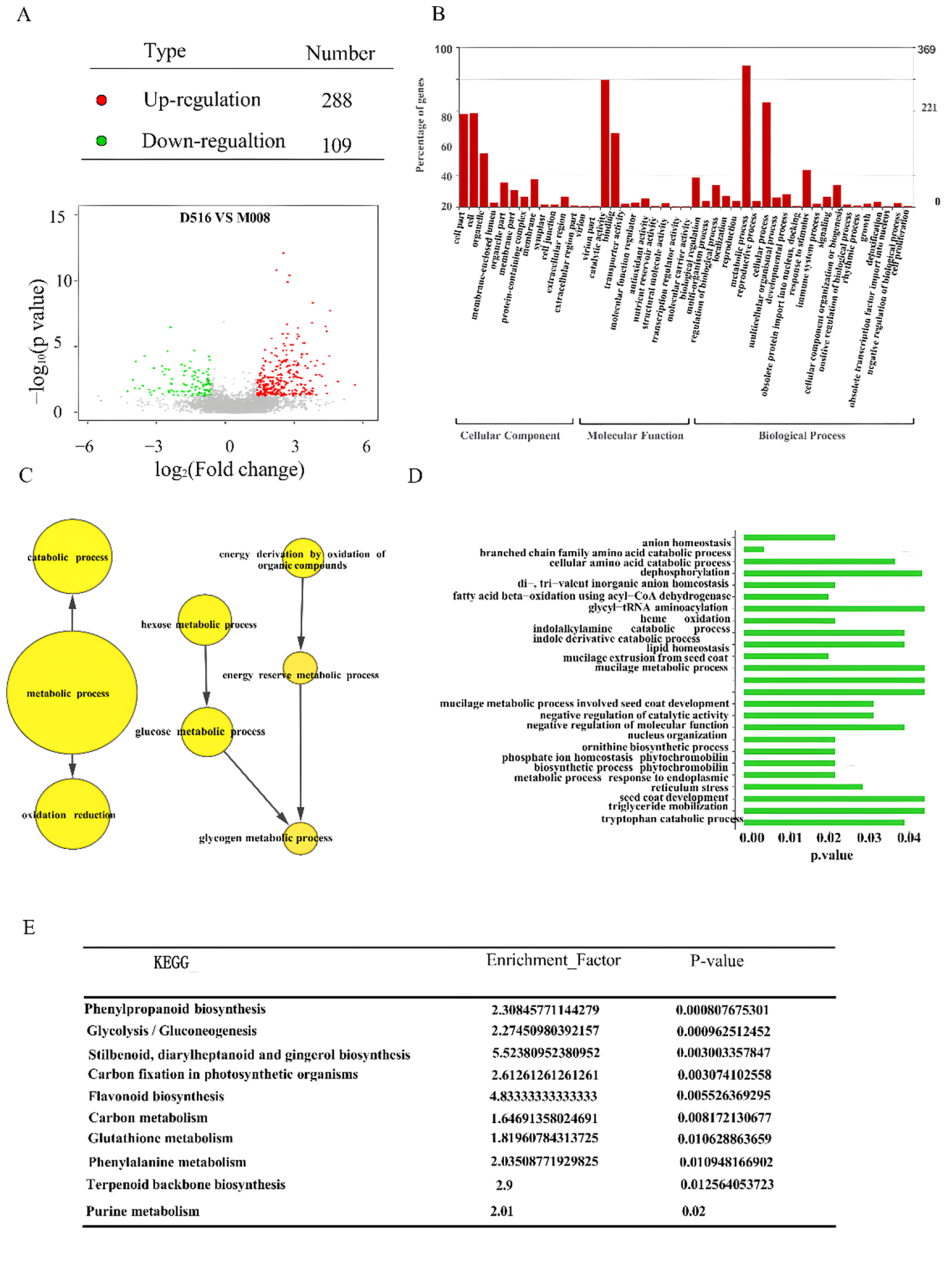
Identification of DAPs between the two potato cultivars. Volcano plot shows the distribution of DAPs between the ‘D516’ and ‘M5008’ cultivars **(A)**; GO classification of DAPs between the two potato cultivars. All DAPs were classified by three GO terms: biological processes, cellular components and molecular function **(B)**; GO enrichment analysis of the DAPs based on GO terms **(C, D)**; KEGG enrichment analysis of all identified DAPs **(E)**.

To identify the DAPs between two potato cultivars, the detected proteins were further analyzed. A total of 397 DAPs were identified, based on combination fold change (≥ 1.5 or ≤ 0.66) and P values (< 0.05), including 288 proteins that were up-regulated and 109 proteins that were down-regulated significantly in the ‘M5008’ cultivar compared to the ‘D516’ cultivar (**Figure 5A**).

The functions of all 397 DAPs were classified into three different categories using gene ontology (GO) classification, with 14 terms in the cellular component categories, 16 terms in the molecular function categories, and 19 terms in the biological process category (**Figure 5B**). In cellular component categories, cell part (58 DAPs), cell (58 DAPs), organelle (33 DAPs), membrane (17 DAPs), and membrane part (10 DAPs) were the top six terms. Catalytic activity (79 DAPs), binding (46 DAPs), and biological regulation (18 DAPs) were the top three terms in molecular function categories. Metabolic process (88 DAPs), cellular process (65 DAPs), response to stimulus (23 DAPs), and cellular component organization or biogenesis (13 DAPs) were the top four terms in biological process categories (**Figure 5B**).

GO annotation identified that 60 DAPs associated with oxidation reduction and 22 DAPs associated with glucose metabolic processes in the ‘M5008’ cultivar were significantly higher than those in the ‘D516’ cultivar (**Figure 5C**). Among these DAPs, Q6R608 (annotated as 4-alpha-glucanotransferase) and Q9FEN7 (annotated as dihydrolipoyl dehydrogenase) proteins in M5008 were 5.9-fold and 4.3-fold compared to D516, respectively (**Supplementary Table S2**). However, six DAPs associated with negative regulation of catalytic activity in the ‘M5008’ cultivar were remarkably lower than that in the ‘D516’ cultivar (**Figure 5D**). The expression of P20347 protein (annotated as cysteine protease inhibitor 1) was 2.9-fold compared to the ‘D516’ cultivar (**Supplementary Table S2**). These results suggested that metabolic differentiation is one of the reasons for salt-tolerance differentiation between ‘M5008’ and ‘D516’ cultivars.

In addition, all DAPs between two potato cultivars were further grouped using KEGG pathway annotation and enrichment analysis (**Figure 5E**). The result showed that nine pathways were remarkably enriched with DAPs, including phenylpropanoid biosynthesis, glycolysis/gluconeogenesis, stilbenoid, diarylheptanoid, and gingerol biosynthesis, carbon fixation in photosynthetic organisms, flavonoid biosynthesis, carbon metabolism, glutathione metabolism, phenylalanine metabolism, and terpenoid backbone biosynthesis. For example, K7WNY2 in the ‘M5008’ cultivar, coding glyceraldehyde-3-phosphate dehydrogenase involved in plant development, provided substrates for the phosphorylated pathway of serine biosynthesis (Munoz-Bertomeu et al., 2009) and had a higher expression than that in the ‘D516’ cultivars.

### 3.3 Identification and functional annotation of DAPs between the two potato cultivars response to salt stress

To determine DAPs in these two potato cultivars response to salt stress, three independent samples of ‘D516’ and ‘M5008’ cultivars with salt-treated (0.15 M NaCl for 12 h) and mock-treated were collected for protein sequencing using comparative iTRAQ analysis. For the ‘D516’ cultivar, a total of 511 DAPs, including 249 proteins with increased expression and 262 proteins with reduced expression, were defined in response to salt treatment. While, for the ‘M5008’ cultivar, a total of 456 DAPs, including 95 proteins with increased expression and 361 proteins with reduced expression, were identified after being exposed to salt conditions (**Figure 5A**; **Supplementary Table S3**).

To deduce the biological processes and mechanisms associated with salt stress in two potato cultivars, GO enrichment and KEGG pathway analysis were further performed. During abiotic stress conditions, hydrogen peroxide is produced and plays a crucial role in acclimating plants to the stress condition (Choudhury et al., 2017). According to the GO analysis result, the signal pathway associated with hydrogen peroxide had been activated in the ‘D516’ cultivar (**Figure 5B**). For example, the expression of M0ZNG4, M1CCK1, and M1C911, which are annotated as peroxidase, were upregulated to 6.2, 10, and 10.6 times in the ‘D516’ cultivars after being exposed to salt conditions (**Figures 5C, D**; **Supplementary Table S3**), while these proteins in the ‘M5008’ cultivar only showed a slight difference. These results suggested that after being exposed to salt conditions for 12 h, excess ROS was accumulated in the ‘D516’ cultivar, which might contribute to oxidative stress damaging plant growth and development. For the ‘M5008’ cultivar, perhaps redox homeostasis was maintained by the enzymatic components and the nonenzymatic compounds, including glutathione metabolism (**Figure 5B**; **Supplementary Table S3**) and vitamin (**Figure 5C**; **Supplementary Table S3**).

Sugars and proteins have multiple roles under salt stress, such as structural components, osmolytes, signaling molecules, and supplements of energy (Massa and Melito, 2019). The GO analysis results showed that many DAPs in D516 were enriched in response to sugar signaling, including response to fructose, sucrose, hexose, monosaccharide, glucose, and disaccharide stimulus (**Figures 5B, D**; **Supplementary Table S3**), suggesting that sugar stimulus activated downstream target gene expression to acclimatize salt stress. However, for M5008, the DAPs were enriched into sugar metabolic and catabolic processes and energy metabolic processes, such as glucose catabolic and metabolic processes, hexose biosynthetic and metabolic processes, and NADP metabolic processes (**Figures 5B, D**; **Supplementary Table S3**). These processes might provide energy and components to acclimate plants to salt conditions, such as maintaining redox homeostasis. Besides, soluble sugar could also be produced through these processes to maintain cellular osmotic pressure. According to the KEGG pathway analysis, protein biosynthesis and metabolism were found in both M5008 and D516, including glycine, serine, threonine, proline, tryptophan, glutathione, and lysine (**Figures 5C, D**; **Supplementary Table S3**). These amino acid metabolisms may provide the components and soluble protein content to protect the tolerant cells.

Auxin is required to mediate plants responses to salt stress (Ribba et al., 2020). Many genes involved in auxin biosynthesis were induced by salt treatment, and auxin signaling also functions in tolerance to salinity stress (Ribba et al., 2020; Verma et al., 2022). GO analysis of M5008 showed that many DAPs were enriched into indolalkylamine biosynthetic processes and auxin-mediated signaling pathways (**Figure 5C**; **Supplementary Table S3**), suggesting that auxin biosynthesis and signaling pathways might be a strategy for the ‘M5008’ cultivar to enhance tolerance to salinity stress.

### 3.4 Analysis for the interaction of DAPs response to salt stress

**Figure 6:**
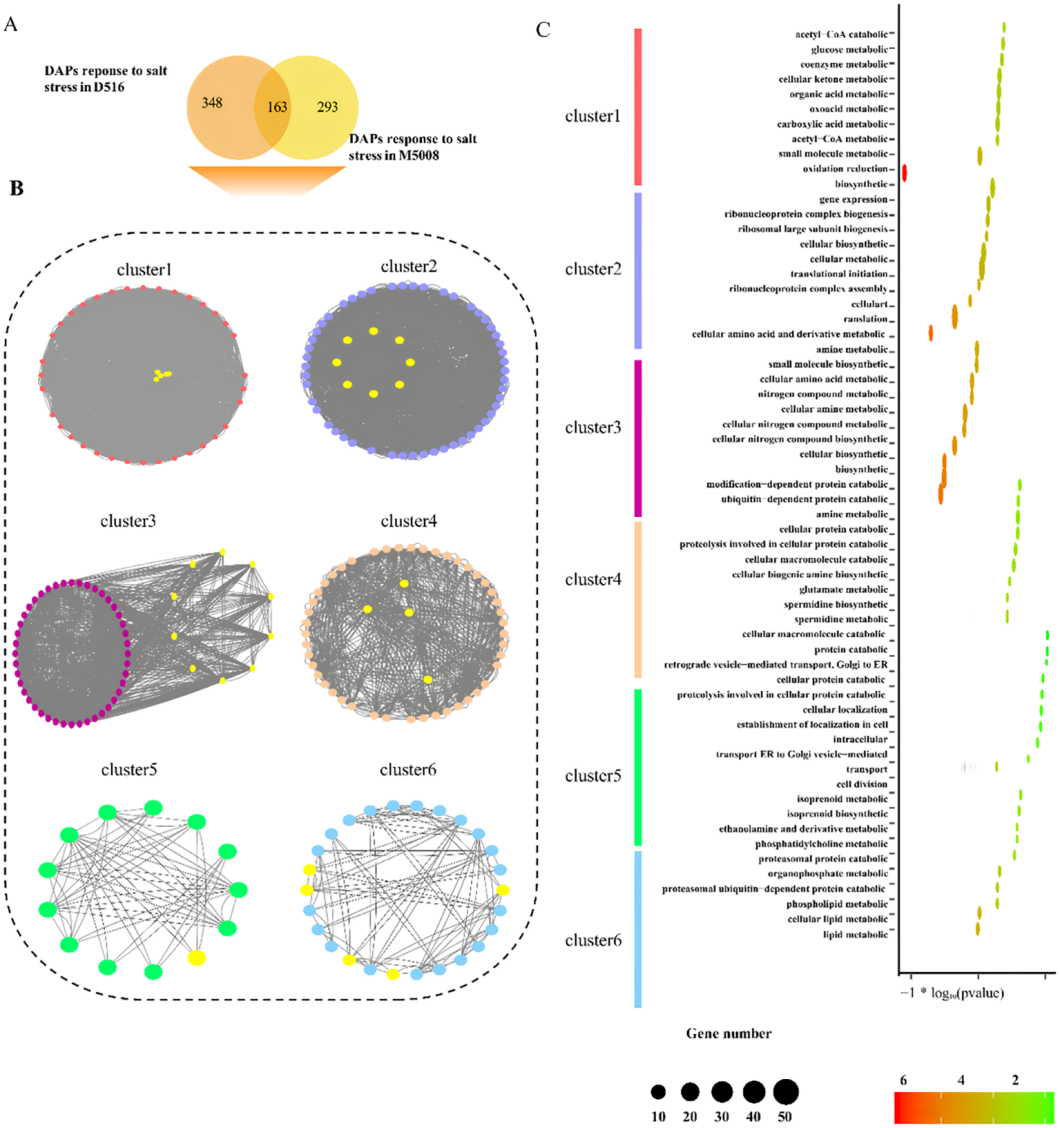
Analysis of the protein interaction between the DAPs response to salt stress. The Venn diagram indicates the shared proteins of DAPs between two potato cultivars **(A)**. Six clusters were identified by the MCODE of Cytoscape from the protein-protein interaction network constructed by DAPs in response to salt stress in the two potato cultivars. Color nodes and edges represented different expressed proteins and the interaction between proteins. Yellow nodes indicate proteins present a similarly expressed pattern both in ‘D516’ and ‘M5008’ cultivars after salt treatment **(B)**. GO enrichment for six clusters. The top 10 GO categories of each cluster are displayed **(C)**.

To further explore the potential and functional modules under salt conditions in potato, all different expressed proteins induced by salt treatment, comprised by 511 and 456 in both ‘D516’ and ‘M5008’ cultivars (**Figure 6A**), were superimposed into protein-protein interaction (PPI) network from the STRING and then identified functional cluster using the MCODE (Bader and Hogue, 2003) of Cytoscape. In total, the largest six distinct clusters with at least 10 nodes were identified and indicated with different colors, in which the proteins with similarly consistent trend after salt treatment in D516 and M5008 were labeled with yellow colors (**Figure 6B**).

Cluster 1 contained 40 different expressed proteins in response to salt in the ‘D516’ and ‘M5008’ cultivars. Additionally, five of all proteins displayed a similarly expressed pattern in both cultivars. For instance, the protein M1AZE4, coding glucose-6-phosphate isomerase, which was proven to promote the synthesis of starch in leaves (Yu et al., 2000), was down-regulated after salt treatment in ‘D516’ and ‘M5008’ cultivars (**Figure 6B**; **Supplementary Table S4**). Cluster 2, composed of 59 proteins, was the largest cluster, including 7 proteins, and exhibited low expression in both cultivars after treatment with salt (**Figure 6B**; **Supplementary Table S4**). A striking example is M1CXP4, the glycine cleavage system P protein, which affects cotyledon stage Arabidopsis (Engel et al., 2007). The 10 proteins in ‘D516’ and ‘M5008’ cultivars expressed a similar inclination response to salt stress in cluster 3 with 54 nodes (**Figure 6B**; **Supplementary Table S4**). For example, the up-regulated protein M1D2W7 coding nucleoside diphosphate kinase 1 has been reported to play a role in response to reactive oxygen species (ROS) stress (Fukamatsu et al., 2003). The 50, 13, and 26 different expressed proteins were constructed in clusters 4, 5, and 6, respectively, in which 4, 1, and 5 proteins in D516 and M5008 appeared in the expression pattern after treatment (**Figure 6B**; **Supplementary Table S4**). Of these 10 similarly expressed proteins, M1AJK1 provided electron transfer to heme oxygenase and cytochrome B5 (Louërat-Oriou et al., 1998) by coding NADPH-cytochrome P450 reductase.

On the other hand, GO enrichment analysis for the six clusters applying the biological process terms was performed. The different specific biological functions were enriched in each cluster, such as glucose metabolic (Cluster 1), translational initiation (Cluster 2), small molecule biosynthetic (Cluster 3), ubiquitin-dependent protein catabolic (Cluster 4), intracellular transport (Cluster 5), and phosphatidylcholine metabolic (Cluster 6) (**Figure 6C**). It was interesting that Cluster 1 also enriched oxidation reduction, consistent with a previous study that found salt stress could alter the expression of several ROS-scavenging enzymes and ROS-responsive regulatory genes (Rodrigo-Moreno et al., 2013). Taken together, these results suggest that the salt-related pathway in D516 and M5008 might be a concordantly regulated network of multiple proteins using interactions between proteins and functional units at a molecular level.

### 3.5 qRT-PCR analysis of candidate proteins response to salt stress and Potato seedling response signals under salinity

**Figure 7:**
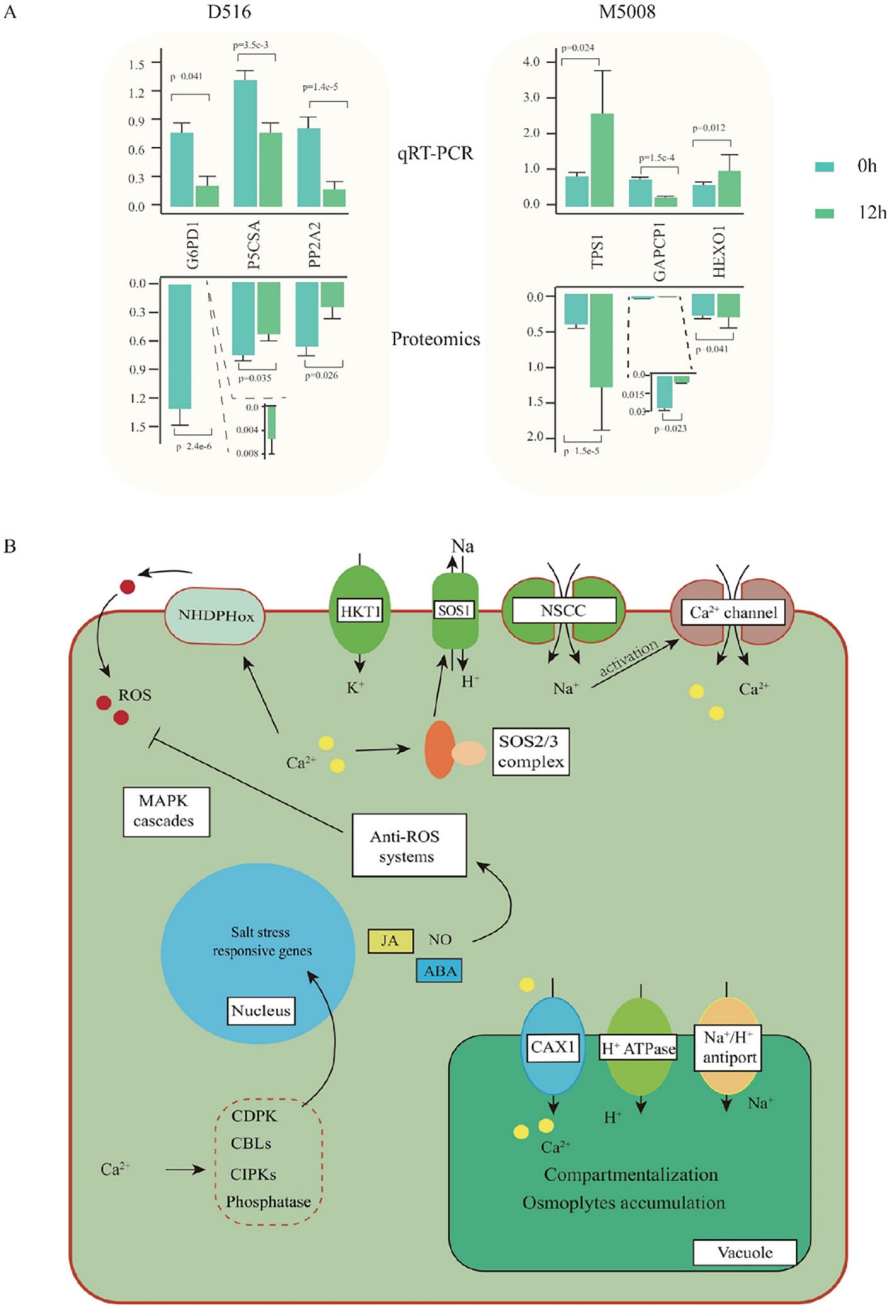
qPCR validation and overview of salinity-regulated networks in potatoes. qCR validation of six proteins at mRNA levels. The up- and down-bar plot indicated the expressed pattern from G6PD1, Glucose-6-phosphate 1-dehydrogenase 1; P5CSA, Delta-1-pyrroline-5-carboxylate synthase A; PP2A2; Serine/threonine-protein phosphatase PP2A-2 catalytic subunit; TPS1, α-trehalose-phosphate synthase; GAPCP1, Glyceraldehyde-3-phosphate dehydrogenase; HEXO, β-hexosaminidase 1 **(A)**; The overview of salinity-regulated networks in potatoes **(B)**.

To determine if the candidate DAPs were associated with their transcriptional alteration, qRT-PCR analysis was performed. The results showed that the gene expression levels of *G6PD1*, *P5CSA*, and *PIP2A2* in the ‘D516’ cultivar were down-regulated significantly after salt treatment. In the ‘M5008’ cultivar, two genes, *TPS1* and *HEXO1*, were up-regulated and one gene, *GAPCP1*, was down-regulated in response to salt stress (**Figure 7A**). Further analysis indicated that the abundance of these six candidate proteins was consistent with their transcription level, indicating a good correlation between the transcription level and the corresponding protein abundance in ‘D516’ and ‘M5008’ cultivars.

**Figure 8:**
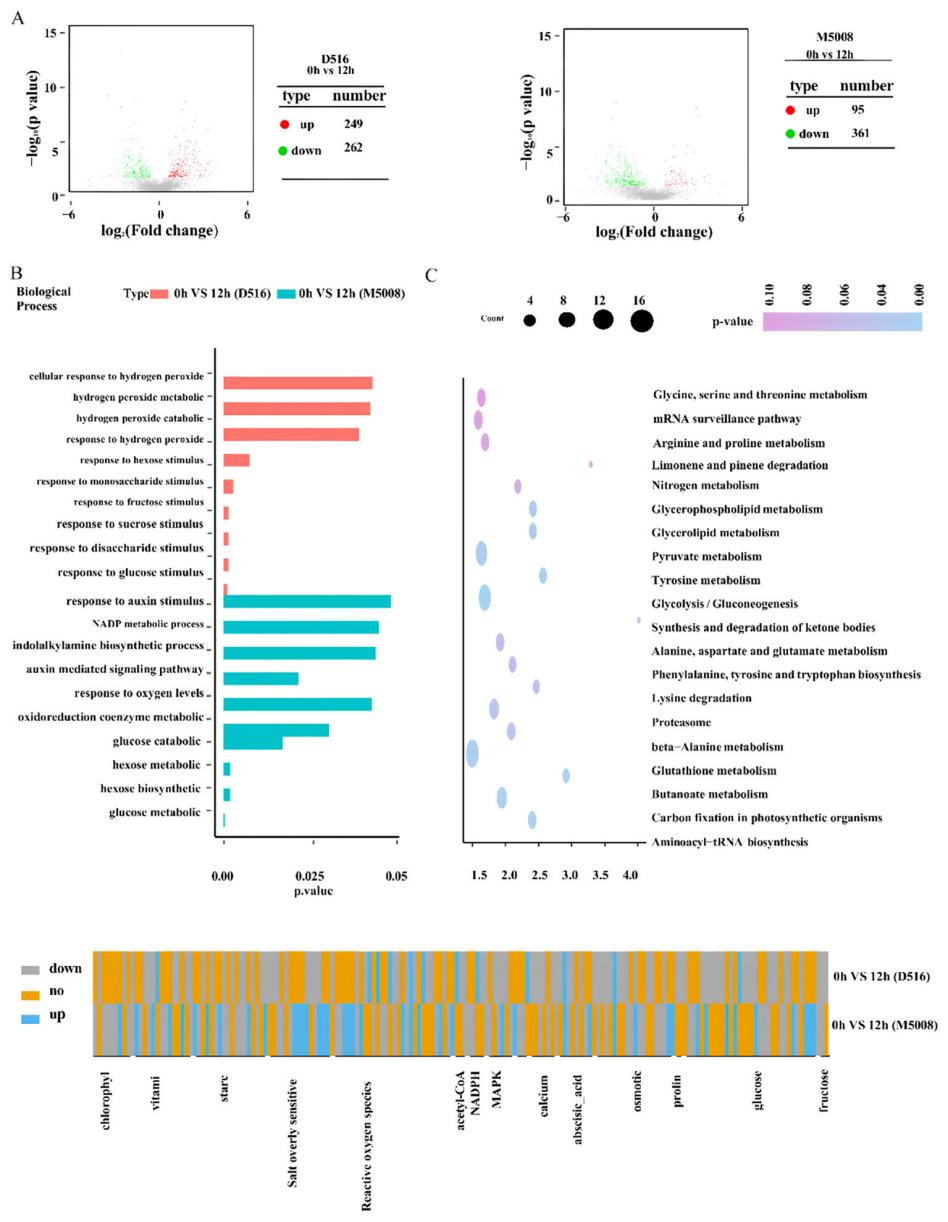
Identification of salt stress-responsive proteins in the two potato cultivars. Volcano plot shows the up- and down-regulated proteins response to salt stress in the ‘D516’ and ‘M5008’ cultivars **(A)** G enrichment analysis for DAPs of the two potato cultivars response to salt stress and the top 10 GO categories of each cultivar are displayed **(B)** KEGG enrichment analysis for DAPs of these two potato cultivars response to salt stress and the top 10 KEGG categories of each cultivar are displayed **(C)** Heat map of log2 (fold changes) for DAPs associated with MAPK, calcium, abscisic acid, chlorophyll, osmotic pathway, glucose metabolism, salt overly sensitive, reactive oxygen species, vitamin metabolism, and starch metabolism in the two potato cultivars response to salt stress **(D)**.

Osmotic stress and ionic toxicity are the main primary effects of salt stress (Arif et al., 2020). Under salinity, Na^+^ accumulates promptly in tolerant cell cytosols through the non-selection cation channel (NSCCs) and K^+^ transporters (HKT1), with a consequent activation of the Ca^2+^ channel (Massa and Melito, 2019). To exclude Na^+^ from the cytosol to the extracellular, the cytosol Ca^2+^ activates the SOS signaling pathway, which is indeed one of the crucial mechanisms to counteract the injurious effects of ion toxics (Köster et al., 2019). SOS3 recognizes the salt-induced Ca^2+^ signal and activates and recruits SOS2 to the cytomembrane, causing the stimulation of the cytomembrane Na^+^/H^+^ antiport (SOS1) activity and further excreting the excess Na^+^ out of the cells (Ishitani et al., 2000). In our study, many genes involved in the SOS pathway were remarkably up-regulated in the ‘M5008’ cultivar but down-regulated in the ‘D516’ cultivar (**Figure 8D**). Additionally, numerous genes involved in the synthesis and metabolism of sugar, such as glucose and hexose (**Figures 8B, D**), were significantly activated in response to salt stress in the ‘M5008’ cultivar, contributing to the adjustment of osmotic stress and the demand for energy and components used for salt response. These findings suggested that the ‘M5008’ cultivar possessed a higher ability to maintain ion homeostasis and adjust osmotic stress.

Oxidative stress is one of the secondary effects of salt stress (Zulfiqar and Ashraf, 2021). Under salinity conditions, the cytosolic Ca^2+^ activates NADPH oxidase, producing a high level of apoplastic ROS (Kurusu et al., 2015). The ROS influx into the cytoplasm is perceived by the membrane-localized histidine kinase, inducing the activation of the MAPK signaling cascade and further regulating the growth and development of the whole plant system (Massa and Melito, 2019). According to our study, the synthesis and signal pathway of ROS were significantly activated in the ‘D516’ cultivar (**Figure 8B**). Redox homeostasis is maintained by enzymatic components, such as SOD, CAT, and glutathione-S-transferase, and nonenzymatic molecular compounds, including proline, GSH, and α-tocopherol (Rajput et al., 2021). From our proteomic data, numerous genes in response to oxygen metabolism and genes involved in the oxidoreduction coenzyme metabolic process in the ‘M5008’ cultivar were activated (**Figure 7B**), maintaining the redox homeostasis in the ‘M5008’ cultivar.

Overall, under salinity conditions, the ‘M5008’ cultivar was endowed with a higher capacity than that in the ‘D516’ cultivar for sensing a saline environment and prompting a reaction to change its physiological status, including ionic equilibrium, osmoregulation, and redox homeostasis maintenance (**Figure 7B**).

## 4 Discussion

This study presents a comprehensive multi-omics perspective on the molecular mechanisms underlying salt tolerance in potato. By integrating physiological, proteomic, and transcriptional data, we demonstrate that the tolerant cultivar ‘M5008’ employs a coordinated, multi-layered strategy to cope with salinity stress, in stark contrast to the disorganized and inefficient response observed in the sensitive ‘D516’.

### 4.1 Proactive antioxidant defense and metabolic homeostasis are hallmarks of tolerance

Our physiological results clearly show that ‘M5008’ maintains a more robust and stable antioxidant system (**Figure 2**). The proteomic data provide a mechanistic basis for this observation: while ‘D516’ exhibited a high-amplitude but transient induction of specific peroxidases (**Figures 8B, D**), ‘M5008’ displayed constitutive and stress-induced enrichment of a broad suite of redox homeostasis components. This included not only enzymes such as SOD and POD but also pathways such as glutathione metabolism (**Figures 5E**; **8C**). This proactive, multi-component strategy likely prevents the damaging accumulation of reactive oxygen species (ROS), as evidenced by the controlled MDA dynamics in ‘M5008’ (**Figure 2D**). These findings align with the emerging consensus that salt tolerance is determined not merely by ROS scavenging capacity but by the ability to maintain redox signaling and homeostasis under stress (Choudhury et al., 2017; Kesawat et al., 2023). Furthermore, the significant enrichment of DAPs in central carbon metabolism—particularly glycolysis and gluconeogenesis—and energy-related processes (**Figure 5E**) in ‘M5008’ suggests a strategic metabolic reprogramming to generate ATP, NADPH, and carbon skeletons essential for osmotic adjustment and the biosynthesis of protective compounds (Zhang et al., 2023).

### 4.2 Precision in protein management and hormonal signaling

PPI network analysis revealed that salt tolerance involves highly organized functional modules. The identification of “translational initiation” (Cluster 2) and “ubiquitin-dependent protein catabolic process” (Cluster 4) as key clusters (**Figures 6B, C**) indicates that ‘M5008’ precisely regulates its proteome. It can selectively translate stress-responsive mRNAs while simultaneously degrading damaged or regulatory proteins via the ubiquitin-proteasome system—a critical mechanism for cellular maintenance under stress, as highlighted in recent studies on plant protein turnover (Wang et al., 2024). Additionally, the enrichment of “auxin-mediated signaling pathways” in ‘M5008’ (**Figure 8B**) implicates phytohormone regulation in its adaptive strategy. Auxin acts as a central regulator of root architecture and growth under stress, and its role in salt tolerance is supported by evidence showing that auxin signaling mutants often exhibit increased sensitivity to salinity (Ribba et al., 2020., Verma et al., 2022).

### 4.3 Membrane remodeling and integrated stress sensing

The enrichment of “phosphatidylcholine metabolic process” in Cluster 6 (**Figure 6C**) underscores the importance of membrane lipid remodeling in salt tolerance. Adjustments in phospholipid composition are crucial for maintaining membrane fluidity and functionality under stress and may contribute to the generation of lipid-derived signaling molecules (Hou et al., 2023) The more coordinated response of ‘M5008’, evident from the tighter clustering of its biological replicates in the PCA plot (**Figure 4**), suggests superior integration of stress sensing and signaling, leading to a consistent and effective proteomic response.

### 4.4 A convergent model for salt tolerance in potato

In conclusion, our findings support a model in which the salt tolerance of ‘M5008’ is conferred by the integrated operation of several key mechanisms (**Figure 7B**):

① Energetic and Metabolic Flexibility: Redirection of carbon flux to fuel defense responses and osmotic adjustment.

② Robust Redox Management: Utilization of a balanced, multi-component antioxidant system to prevent oxidative damage.

③ Proteomic Precision: Coordinated regulation of protein synthesis and degradation to maintain proteome integrity and adaptability.

④ Enhanced Signaling and Infrastructure: Engagement of hormone signaling (e.g., auxin) and membrane remodeling to facilitate adaptive growth and preserve cellular integrity.

## 5 Conclusion

In contrast, ‘D516’ lacks this coordination, resulting in a dysregulated, metabolically inefficient, and ultimately ineffective stress response. The proteins and functional modules identified in this study—particularly those validated by qRT-PCR, such as TPS1 (involved in trehalose synthesis for osmoprotection) and the redox-associated G6PD1—provide a valuable resource of candidate targets for future functional characterization and for molecular breeding and genetic engineering of salt-tolerant potato varieties.

## Data Availability

The original contributions presented in the study are included in the article/**Supplementary Material**. Further inquiries can be directed to the corresponding author.

## Supplementary material

Supplementary Table 1 Statistics of all proteins from iTRAQ method.

Supplementary Table 2 Differentially accumulated proteins (DAPs) between two potato cultivars.

Supplementary Table 3 Differentially accumulated proteins (DAPs) from the two potato cultivars response to salt stress.

Supplementary Table 4 Clusters from the interaction of DAPs in response to salt stress.

Supplementary Table 5 Primers used for qRT-PCR.

## References

[B1] Abbasi-Vineh M. A. Sabet M. S. Karimzadeh G. (2021). Identification and functional analysis of two purple acid phosphatases AtPAP17 and AtPAP26 involved in salt tolerance in Arabidopsis thaliana plant. Front. Plant Sci. 11, 618716. doi: 10.3389/fpls.2020.618716, PMID: 33679819 PMC7928345

[B2] Ahmad R. Hussain S. Anjum M. A. Khalid M. F. Saqib M. Zakir I. . (2019). “ Oxidative stress and antioxidant defense mechanisms in plants under salt stress.” In Plant Abiotic Stress Tolerance: Agronomic, Molecular, and Biotechnological Approaches (Cham: Springer), pp. 191–205. doi: 10.1007/978-3-030-06118-08

[B3] Anders S. Huber W. (2010). Differential expression analysis for sequence count data. Genome Biol. 11, R106. doi: 10.1186/gb-2010-11-10-r106, PMID: 20979621 PMC3218662

[B4] Arif Y. Singh P. Siddiqui H. Bajguz A. Hayat S. (2020). Salinity induced physiological and biochemical changes in plants: An omic approach towards salt stress tolerance. Plant Physiol. Biochem. 156, 64–77. doi: 10.1016/j.plaphy.2020.08.042, PMID: 32906023

[B5] Ashburner M. Ball C. A. Blake J. A. Botstein D. Butler H. Cherry J. M. . (2000). Gene ontology: tool for the unification of biology. Nat. Genet. 25, 25–29. doi: 10.1038/75556, PMID: 10802651 PMC3037419

[B6] Aslam M. Ahmad K. Akhtar M. A. Maqbool M. A. (2019). “ Oxidative Stress and Antioxidant Defense Mechanisms in Plants Under Salt Stress,” in Plant Abiotic Stress Tolerance. Eds. Hasanuzzaman M. Hakeem K. Nahar K. Alharby H. ( Springer, Cham).

[B7] Bader G. D. Hogue C. W. (2003). An automated method for finding molecular complexes in large protein interaction networks. BMC Bioinf. 4, 1–27. doi: 10.1186/1471-2105-4-2, PMID: 12525261 PMC149346

[B8] Bairoch A. Apweiler R. (2000). The SWISS-PROT protein sequence database and its supplement TrEMBL in 2000. Nucleic Acids Res. 28, 45–48. doi: 10.1093/nar/28.1.45, PMID: 10592178 PMC102476

[B9] Balasubramaniam T. Shen G. Esmaeili N. Zhang H. (2023). Plants’ response mechanisms to salinity stress. Plants 12, 2253. doi: 10.3390/plants12122253, PMID: 37375879 PMC10300796

[B10] Choudhury F. K. Rivero R. M. Blumwald E. Mittler R. (2017). Reactive oxygen species, abiotic stress and stress combination. Plant J. 90, 856–867. doi: 10.1111/tpj.13299, PMID: 27801967

[B11] Devaux A. Goffart J. P. Kromann P. Andrade-Piedra J. Polar V. Hareau G. (2021). The potato of the future: opportunities and challenges in sustainable agri-food systems. Potato Res. 64, 681–720. doi: 10.1007/s11540-021-09501-4, PMID: 34334803 PMC8302968

[B12] Dos Santos T. B. Ribas A. F. de Souza S. G. H. Budzinski I. G. F. Domingues D. S. (2021). Physiological responses to drought, salinity, and heat stress in plants: a review. Stresses 2, 113–135. doi: 10.3390/stresses2010009

[B13] Dzinyela R. Alhassan A. R. Suglo P. Movahedi A. (2023). Advanced study of functional proteins involved in salt stress regulatory pathways in plants. S. Afr. J. Bot. 159, 425–438. doi: 10.1016/j.sajb.2023.06.029

[B14] El-Gebali S. Mistry J. Bateman A. Eddy S. R. Luciani A. Potter S. C. . (2019). The Pfam protein families database in 2019. Nucleic Acids Res. 47, 427–432. doi: 10.1093/nar/gky995, PMID: 30357350 PMC6324024

[B15] Engel N. van den Daele K. Kolukisaoglu U. Morgenthal K. Weckwerth W. Pärnik T. . (2007). Deletion of glycine decarboxylase in Arabidopsis is lethal under nonphotorespiratory conditions. Plant Physiol. 144, 1328–1335. doi: 10.1104/pp.107.099317, PMID: 17496108 PMC1914133

[B16] Fan H. Xu Y. Du C. Wu X. (2015). Phloem sap proteome studied by iTRAQ provides integrated insight into salinity response mechanisms in cucumber plants. J. Proteomics 125, 54–67. doi: 10.1016/j.jprot.2015.05.001, PMID: 25958826

[B17] Fu H. Yang Y. (2023). How plants tolerate salt stress. Curr. Issues Mol. Biol. 45, 5914–5934. doi: 10.3390/cimb45070374, PMID: 37504290 PMC10378706

[B18] Fukamatsu Y. Yabe N. Hasunuma K. (2003). *Arabidopsis* NDK1 is a component of ROS signaling by interacting with three catalases. Plant Cell Physiol. 44, 982–989. doi: 10.1093/pcp/pcg140, PMID: 14581623

[B19] Han Y. Yin S. Huang L. (2015). Towards plant salinity tolerance-implications from ion transporters and biochemical regulation. Plant Growth Regul. 76, 13–23. doi: 10.1007/s10725-014-9997-6

[B20] Hasanuzzaman M. Raihan M. R. H. Masud A. A. C. Rahman K. Nowroz F. Rahman M. . (2021). Regulation of reactive oxygen species and antioxidant defense in plants under salinity. Int. J. Mol. Sci. 22, 9326. doi: 10.3390/ijms22179326, PMID: 34502233 PMC8430727

[B21] Hou Q. Zhang S. Wang L. (2023). The role of phospholipid signaling in plant salt stress response. Front. Plant Science. 14, 1156874. doi: 10.3389/fpls.2023.1156874

[B22] Ishitani M. Liu J. Halfter U. Kim C. S. Shi W. Zhu J. K. (2000). SOS3 function in plant salt tolerance requires N-myristoylation and calcium binding. Plant Cell 12, 1667–1677. doi: 10.1105/tpc.12.9.1667, PMID: 11006339 PMC149077

[B23] Kanehisa M. Goto S. Kawashima S. Nakaya A. (2002). The KEGG databases at GenomeNet. Nucleic Acids Res. 30, 42–46. doi: 10.1093/nar/30.1.42, PMID: 11752249 PMC99091

[B24] Kesawat M. S. Satheesh N. Kherawat B. S. Kumar A. Kim H. U. Chung S. M. . (2023). Regulation of reactive oxygen species during salt stress in plants and their crosstalk with other signaling molecules - Current perspectives and future directions. Plants 12, 864. doi: 10.3390/plants12040864, PMID: 36840211 PMC9964777

[B25] Köster P. Wallrad L. Edel K. H. Faisal M. Alatar A. A. Kudla J. (2019). The battle of two ions: Ca^2+^ signalling against Na^+^ stress. Plant Biol. 21, 39–48. doi: 10.1111/plb.12859, PMID: 29411929

[B26] Kurusu T. Kuchitsu K. Tada Y. (2015). Plant signaling networks involving Ca^2+^ and Rboh/Nox-mediated ROS production under salinity stress. Front. Plant Sci. 6, 145821. doi: 10.3389/fpls.2015.00427, PMID: 26113854 PMC4461821

[B27] Lakra N. Kaur C. Singla-Pareek S. L. Pareek A. (2019). Mapping the ‘early salinity response’ triggered proteome adaptation in contrasting rice genotypes using iTRAQ approach. Rice 12, 1–22. doi: 10.1186/s12284-018-0259-5, PMID: 30701331 PMC6357216

[B28] Louërat-Oriou B. Perret A. Pompon D. (1998). Differential redox and electron-transfer properties of purified yeast, plant and human NADPH-cytochrome P-450 reductases highly modulate cytochrome P-450 activities. Eur. J. Biochem. 258, 1040–1049., PMID: 9990323 10.1046/j.1432-1327.1998.2581040.x

[B29] Maere S. Heymans K. Kuiper M. (2005). BiNGO: a Cytoscape plugin to assess overrepresentation of gene ontology categories in biological networks. Bioinformatics 21, 3448–3449. doi: 10.1093/bioinformatics/bti551, PMID: 15972284

[B30] Massa D. Melito S. (2019). “ Signaling molecules in ecophysiological response mechanisms of salt-stressed plants.” Plant Signaling Molecules: Role and Regulation under Stressful Environments. Khan M. I. R. Reddy P. S. Ferrante A. Khan N. A. (Eds.). ( Woodhead Publishing). pp. 265–280. doi: 10.1016/B978-0-12-816451-8.00016-6

[B31] Massange-Sánchez J. A. Sánchez-Hernández C. V. Hernández-Herrera R. M. Palmeros-Suárez P. A. (2021). “ The Biochemical Mechanisms of Salt Tolerance in Plants,” in Plant Stress Physiology-Perspectives in Agriculture ( IntechOpen, London, UK).

[B32] Munoz-Bertomeu J. Cascales-Minana B. Mulet J. M. Baroja-Fernández E. Pozueta-Romero J. Kuhn J. M. . (2009). Plastidial glyceraldehyde-3-phosphate dehydrogenase deficiency leads to altered root development and affects the sugar and amino acid balance in Arabidopsis. Plant Physiol. 151, 541–558. doi: 10.1104/pp.109.143701, PMID: 19675149 PMC2754643

[B33] Peng Z. He S. Sun J. Pan Z. Gong W. Lu Y. . (2016). Na^+^ compartmentalization related to salinity stress tolerance in upland cotton (*Gossypium hirsutum*) seedlings. Sci. Rep-UK 6, 34548. doi: 10.1038/srep34548, PMID: 27698468 PMC5048304

[B34] Pruitt K. D. Tatusova T. Maglott D. R. (2007). NCBI reference sequences (RefSeq): a curated non-redundant sequence database of genomes, transcripts and proteins. Nucleic Acids Res. 35, D61–D65. doi: 10.1093/nar/gkl842, PMID: 17130148 PMC1716718

[B35] Rajput V. D. Harish Singh R. K. Verma K. K. Sharma L. Quiroz-Figueroa F. R. . (2021). Recent developments in enzymatic antioxidant defence mechanism in plants with special reference to abiotic stress. Biology 10, 267. doi: 10.3390/biology10040267, PMID: 33810535 PMC8066271

[B36] Ribba T. Garrido-Vargas F. O’Brien J. A. (2020). Auxin-mediated responses under salt stress: From developmental regulation to biotechnological applications. J. Exp. Bot. 71, 3843–3853. doi: 10.1093/jxb/eraa241, PMID: 32433743

[B37] Rodrigo-Moreno A. Poschenrieder C. Shabala S. (2013). Transition metals: a double edge sward in ROS generation and signaling. Plant Signal. Behav. 8, e23425. doi: 10.4161/psb.23425, PMID: 23333964 PMC3676510

[B38] Ross P. L. Huang Y. N. Marchese J. N. Williamson B. Parker K. Hattan S. . (2004). Multiplexed protein quantitation in Saccharomyces cerevisiae using amine-reactive isobaric tagging reagents. Mol. Cell Proteomics 3, 1154–1169. doi: 10.1074/mcp.M400129-MCP200, PMID: 15385600

[B39] Sobhanian H. Aghaei K. Komatsu S. (2011). Changes in the plant proteome resulting from salt stress: toward the creation of salt-tolerant crops? J. Proteomics 74, 1323–1337. doi: 10.1016/j.jprot.2011.03.018, PMID: 21440686

[B40] Tatusov R. L. Galperin M. Y. Natale D. A. Koonin E. V. (2000). The COG database: a tool for genome-scale analysis of protein functions and evolution. Nucleic Acids Res. 28, 33–36. doi: 10.1093/nar/28.1.33, PMID: 10592175 PMC102395

[B41] Tomar R. S. Kataria S. Jajoo A. (2021). Behind the scene: Critical role of reactive oxygen species and reactive nitrogen species in salt stress tolerance. J. Agron. Crop Sci. 207, 577–588. doi: 10.1111/jac.12490

[B42] Tyanova S. Temu T. Cox J. (2016). The MaxQuant computational platform for mass spectrometry-based shotgun proteomics. Nat. Protoc. 11, 2301–2319. doi: 10.1038/nprot.2016.136, PMID: 27809316

[B43] Verma S. Negi N. P. Pareek S. Mudgal G. Kumar D. (2022). Auxin response factors in plant adaptation to drought and salinity stress. Physiol. Plantarum 174, e13714. doi: 10.1111/ppl.13714, PMID: 35560231

[B44] Wang K. Li Y. Zhang H. (2024). Ubiquitin-mediated protein turnover in plant stress responses: mechanisms and regulation. Trends Plant Sci. 29, 123–135. doi: 10.1016/j.tplants.2023.10.007, PMID: 37827897

[B45] Weng Q. Zhao Y. Yanan Z. Song X. Yuan J. Liu Y. (2021). Identification of salt stress-responsive proteins in maize (*Zea may*) seedlings using iTRAQ-based proteomic technique. Iran. J. Biotechnol. 19, e2512. doi: 10.30498/ijb.2021.2512, PMID: 34179187 PMC8217532

[B46] Wu X. Xiong E. Wang W. Scali M. Cresti M. (2014). Universal sample preparation method integrating trichloroacetic acid/acetone precipitation with phenol extraction for crop proteomic analysis. Nat. Protoc. 9, 362–374. doi: 10.1038/nprot.2014.022, PMID: 24434803

[B47] Yu T. S. Lue W. L. Wang S. M. Chen J. (2000). Mutation of Arabidopsis plastid phosphoglucose isomerase affects leaf starch synthesis and floral initiation. Plant Physiol. 123, 319–326. doi: 10.1104/pp.123.1.319, PMID: 10806248 PMC59005

[B48] Yu G. Wang L. G. Han Y. He Q. Y. (2012). clusterProfiler: an R package for comparing biological themes among gene clusters. Omics: J. Integr. Biol. 16, 284–287. doi: 10.1089/omi.2011.0118, PMID: 22455463 PMC3339379

[B49] Yuan Z. Zhang C. Zhu W. Yan G. Chen X. Qiu P. . (2023). Molecular mechanism that underlies cotton response to salt and drought stress revealed by complementary transcriptomic and iTRAQ analyses. Environ. Exp. Bot. 209, 105288. doi: 10.1016/j.envexpbot.2023.105288

[B50] Zhang H. Zhu J. Gong Z. Zhu J. K. (2023). Metabolic reprogramming in plant abiotic stress response: From osmolyte accumulation to central carbon metabolism. Trends Plant Sci. 28, 1150–1165. doi: 10.1016/j.tplants.2023.07.007, PMID: 37599162

[B51] Zhong C. Q. Li Y. Yang D. Zhang N. Xu X. Wu Y. . (2014). Quantitative phosphoproteomic analysis of RIP3-dependent protein phosphorylation in the course of TNF-induced necroptosis. Proteomics 14, 713–724. doi: 10.1002/pmic.201300326, PMID: 24453211

[B52] Zulfiqar F. Ashraf M. (2021). Nanoparticles potentially mediate salt stress tolerance in plants. Plant Physiol. Biochem. 160, 257–268. doi: 10.1016/j.plaphy.2021.01.028, PMID: 33529801

